# Tertiary Lymphoid Structures in Microorganism-Related Cancer

**DOI:** 10.3390/cancers16203464

**Published:** 2024-10-12

**Authors:** Shuzhe Deng, Xinxin Yang, Lin He, Yunjing Hou, Hongxue Meng

**Affiliations:** 1 Department of Pathology, Harbin Medical University Cancer Hospital, Harbin 150086, China; 3233@hrbmu.edu.cn; 2 Precision Medical Center, Harbin Medical University Cancer Hospital, Harbin 150086, China; 900212@hrbmu.edu.cn (X.Y.); 2019021463@hrbmu.edu.cn (Y.H.); 3 Department of Stomatology, Heilongjiang Provincial Hospital, Harbin 150000, China; j18846719333@163.com

**Keywords:** tertiary lymphoid structures (TLSs), microorganism, cancer

## Abstract

**Simple Summary:**

The role of tertiary lymphoid structures (TLSs) in cancer has been extensively studied, including predicting good prognosis and immunotherapy efficacy. Studies have shown that the induction strategy for the formation mechanism of TLSs is a new direction for tumor therapy, such as tumor vaccines against microorganisms. This article mainly describes the interaction between TLSs and microorganism-related cancer and provides new ideas for the clinical application of TLSs.

**Abstract:**

Tertiary lymphoid structures (TLSs) are ectopic lymphoid tissues formed by the accumulation of lymphocytes and other components outside lymphoid organs. They have been shown to be widespread in cancers and have predictive effects on prognosis and immunotherapy efficacy; however, there is no standardized measurement guide. This paper provides a reference for future research. Moreover, the induction strategy for the formation mechanism of TLSs is a new direction for future cancer treatment, such as cancer vaccines for microorganisms. The effects of microorganisms on cancer are dual. The role of microorganisms, including bacteria, parasites, viruses, and fungi, in promoting cancer has been widely confirmed. However, the specific mechanism of their tumor suppressor effect, particularly the promotion of TLS formation, is currently unknown. In this review, we summarize the role of TLSs in cancer related to microbial infection and provide new ideas for further understanding their mechanisms of action in cancer.

## 1. Introduction

Cancer is a complex process, and therapeutic methods for cancer are also evolving. In recent years, immunotherapy has gained more attention owing to the persistent response of immune checkpoint inhibitors (ICIs) in cancer [1]. However, not all patients benefit from this type of treatment, and more accurate biomarkers are urgently required. The tumor microenvironment (TME) is strongly related to tumor invasion, metastasis, recurrence, and drug resistance [2]. The active infiltration of some tumor-infiltrating lymphocytes (TILs) may reflect a better prognosis and is related to the reaction of the immune checkpoint blockade (ICB) [3,4]. These TILs and stromal cells exist as organized cellular clusters referred to as tertiary lymphoid structures (TLSs), similar to secondary lymphoid organs (SLOs) [5]. The predictive value of TLSs in judging prognosis and evaluating curative effects has been confirmed in several types of solid tumors [6,7,8,9], whereas the discordant state of TLSs in different tumors is attributed to the polymorphism of cell components, structural characteristics, density, location, maturity, and function [10]. There is no complete summary of the detection methods and classification standards.

Research on microorganisms and cancer has also become a hot topic in recent years. Microorganisms have carcinogenic as well as preventive and therapeutic effects, mainly because of their antigenic properties that regulate the host body lymphocytes involved in tumor immunity [11]. In addition, survival and immunotherapy outcomes are better in microbially infected individuals than in uninfected individuals [12]; this may be related to the formation and maturation of TLSs induced by microbial infection.

In this review, we first provide research ideas on TLSs, including how to identify and group them. Second, the current research progress on microorganisms and cancer has been generalized. Finally, the role of TLSs in microorganism-related cancer is summarized, and the mechanism of microorganism-induced TLS formation is discussed. This paper provides a theoretical basis for the microorganism-based TLS induction strategy as a new opportunity for tumor treatment.

## 2. Overview of TLSs

### 2.1. Structure and Composition of TLSs

TLSs are ectopic lymphoid tissues formed by the accumulation of lymphocytes and other components outside lymphoid organs. They do not exist under normal conditions and can be observed in a variety of chronic inflammatory conditions such as autoimmune diseases, infectious diseases, organ transplantation, and cancer [13,14,15].

**Figure 1:**
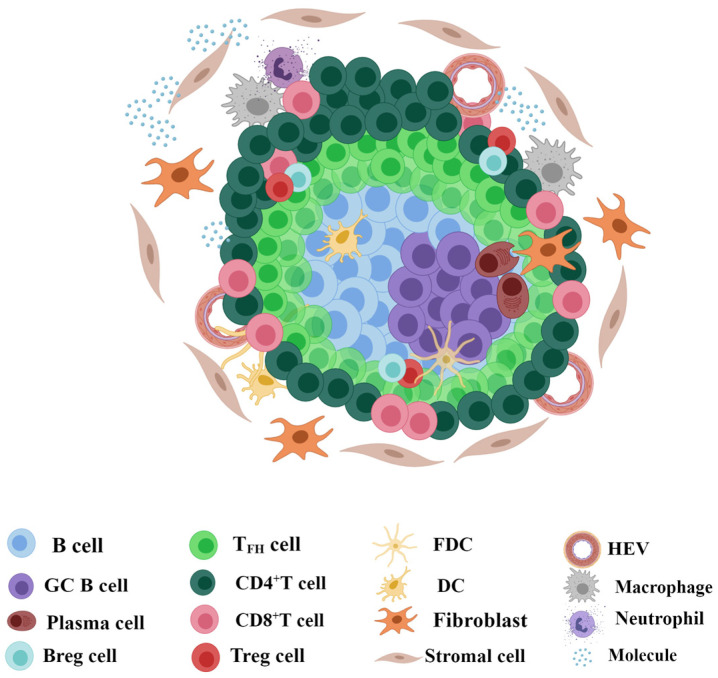
Structure and composition of TLSs. Tertiary lymphoid structures (TLSs) mainly consist of aggregated CD20^+^ B cells as the core and CD3^+^ T cells wrapped around the outside. In the B-cell realm, CD83^+^ dendritic cells (DCs) and CD21^+^ follicular dendritic cells (FDCs) are the most frequent, followed by CD23^+^BCL6^+^ germinal center (GC) B cells and CD38^+^/CD138^+^ plasma cells. In the T-cell realm, CD8^+^ T cells and CD4^+^ T cells containing follicular helper T (T_FH_) cells and regulatory T (Treg) cells are mainly observed, whereas CD19^+^ regulatory B (Breg) cells are observed in some instances. Importantly, high endothelial venules (HEVs) can be observed in the periphery. CD68^+^ macrophages and neutrophils are also scattered in TLSs. Therefore, molecules with important functions should not be ignored. The figure was drawn using the MedPeer program.

TLSs mainly consist of aggregated CD20^+^ B cells as the core, with CD3^+^ T cells wrapped around their outside [15,16,17] (Figure 1). Importantly, the activation and proliferation of Podoplanin (PDPN)-positive immune fibroblasts controlled by interleukin-13 (IL-13) and IL-22 are necessary for the formation of TLSs [18], and the matrix network formed by them provides structural support for TLSs. The peripheral mouse endothelial cell antigen-79 (MECA79)^+^/peripheral node addressin (PNAd)^+^ high endothelial venules (HEVs) allow lymphocyte extravasation due to their irregular cell height and strong deformability and provide the basis for the initiation of TLSs by recruiting lymphocyte aggregates [19]. In addition, CD68^+^ macrophages, interferon regulatory factor 7 (IRF7) nuclear-expressed plasmacytoid dendritic cells (pDCs), and neutrophils are also scattered in TLSs [20,21,22]. It should be emphasized that chemokines and cell molecules with important functions should not be ignored (Table 1).

### 2.2. Identification and Detection of TLSs

TLSs contain several cell types and have complex structures. Identifying and quantifying TLSs in an organization is an urgent problem to be addressed. The common methods used are introduced here (Table 2).

We can identify dense lymphoid tissue aggregates stained with hematoxylin and eosin (HE). Mature TLS-containing germinal centers (GCs) can be clearly defined, and the number, size, morphology, and location of TLSs can be directly observed based on histomorphology; however, the borderline between B-cell zones and T-cell zones cannot be recognized, nor can atypical TLS structures or other non-TLS lymphocyte aggregates be observed. Lymphoid follicles (LFs) are the most easily detected organized TLSs through HE staining. Recently, the rapid development of artificial intelligence and digital pathology provide a highly efficient method for automatic identification and quantification. Barmpoutis et al. observed that the minimal area and minimal number of lymphocytes in TLSs were 6.245 μm^2^ and 45, respectively. The average density of lymphocytes in TLSs was 0.0128 μm^2^, which was approximately three times that of the non-TLS region which was 0.004 μm^2^ [30,31,32].

We can selectively label immune cells within TLSs and analyze their contents and locations using immunohistochemistry (IHC) and immunofluorescence (IF). They both can visually characterize the TLS structure. The tissue sections can be digitized into a whole-slide image (WSI) and analyzed using image analysis software to assess the density, location, and other information of TLSs [33], which provides strong support for studying the interaction between cells. They are the commonly used technologies in TLS research [34]. Flow cytometry (FCM) can be used to separate target components from fresh tissues, verify the results of IHC, and explore the function of the cells by detecting the content of related cytokines [35,36].

Although the most common method to detect TLSs is HE combined with IHC or IF, with the advances in sequencing methods, TLS analysis has accessed the transcriptome level. TLS-related gene signatures can be obtained through RNA sequencing and transcriptome analysis [23,24,28]. For instance, B-cell and chemokine signatures have been detected in Merkel cell carcinoma (MCC) and head and neck squamous-cell carcinoma (HNSCC), respectively [12,37]. Furthermore, a 12-chemokine gene signature in multiple cancers has been confirmed to correlate with survival and ICB efficacy [29,38]. The current signatures of TLSs and their related chemokines are multiple because of tumor and population heterogeneity. Notably, Andersson invented a method for identifying gene expression-based TLSs using a linear regression model that extracts genetic features from model parameters and predicts clinical outcomes based on TLS scores [39].

In conclusion, the detection methods of TLSs in tumors are diverse, and each has advantages and disadvantages. Reasonably integrating these methods and determining an effective measurement method is particularly important, which can ensure that the future application of TLSs in predicting prognosis and judging efficacy will become more standardized.

### 2.3. Classification of TLSs

#### 2.3.1. Presence, Quantity, and Density

**Figure 2:**
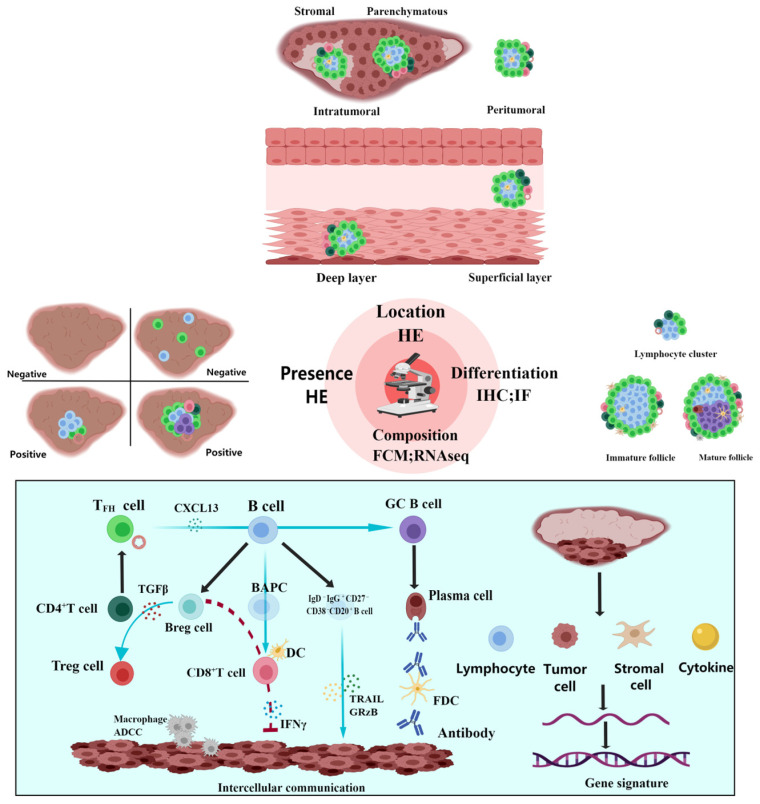
Detection and classification of TLSs. First, it should be determined whether tertiary lymphoid structures (TLSs) exist. TLS-negative status was determined when there were no immune cells in or around the tumor and there were focally distributed immune cells without aggregation. The main detection method of TLSs is hematoxylin and eosin (HE) staining, by which the quantity, density, and location can be observed. There are two types of location-based classification. The first is that TLSs can be divided into intratumoral and peritumoral based on the spatial location, and the former can be further subdivided into parenchymatous and stromal. The second is that TLSs can be divided into superficial and deep layers based on the infiltration location. Combined with immunohistochemistry (IHC) or immunofluorescence (IF) based on HE, TLS differentiation can be divided into lymphocyte cluster, immature follicle, and mature follicle. In addition, TLSs can be studied at the cellular and gene levels using flow cytometry (FCM) and sequencing technology. At present, evidence of intercellular interaction and multiple gene signatures have been obtained. The figure was drawn using the MedPeer program.

It should be determined whether TLSs exist initially (Figure 2); TLS-negative status was determined when there were no immune cells in or around the tumor and there were focally distributed immune cells without aggregation [16]. At present, there is no uniform standard for the definition of TLS positivity; some defined two or more lymphoid aggregates as TLS-positive [40], and some marked lymphocyte aggregation areas greater than 400-fold the microscopic field of view (0.03125 mm^2^) as TLS-positive [41]. In a recently published cohort study, patients with >50 lymphocytes were included in the TLS-positive group [42]. In existing studies, differences in the results of some studies cannot be ruled out because of the non-standardized definition of TLS positivity (Table 3). There are two main methods for grouping TLSs according to quantity. One has been classified into two classes: one or more [43]. The other was classified into three classes: no TLSs (TLS0), one to four TLSs (TLS1–4), and more than five TLSs (TLS≥5) [44]. The results of the former showed that the number of TLSs was related to good prognosis, while the latter showed no correlation. In addition, some scholars have combined the number and size of TLSs as a grouping standard [45]. The density of TLSs and their components can be evaluated using software [42]. The former refers to the number per square millimeter.

#### 2.3.2. Location

First, TLSs can be divided into intratumoral and peritumoral, according to their spatial location [29,41,42,53], and the former can be further subdivided into parenchymatous and stromal [44,54]. It should be emphasized that the definition of the invasive margin of the tumor was either area-based [54] or distance-based [42]. In a study on intrahepatic cholangiocarcinoma, it was found that intratumoral TLSs are associated with a favorable prognosis, whereas peritumoral TLSs are associated with a poor prognosis, indicating that TLSs located in different domains may play a role in tumor suppression or promotion through different immune mechanisms [55]. Second, TLSs can be divided into superficial and deep layers, based on their infiltration location. The former has a more pronounced T helper (Th) cell enrichment and a lower proportion of mature follicles [54].

#### 2.3.3. Differentiation

The development of TLSs has undergone continuous maturation, and the differentiation process of TLSs has been preliminarily defined [15,41,43,46,56]. The initial stage shows lymphocyte clusters containing only T cells, B cells, and HEVs; thus, dendritic cells (DCs) participate to form immature follicles with early structure. Finally, they develop into mature follicles containing follicular dendritic cells (FDCs) and GCs, namely mature TLS. Studies have shown that patients with mature TLS have lower recurrence rates [49,50] and longer survival [42] in certain tumors. Interestingly, only immature TLS was observed in the study of early liver cancer and precancerous lesions, accompanied by overexpression of immunosuppressive molecules [57]. These results indicate that mature TLS has stronger antitumor function.

#### 2.3.4. Composition

The components of TLSs tend to be more refined owing to the popularity of sequencing technology. Programmed cell death protein 1 (PD1)^+^ CD8^+^ T cells [26,53,58,59], PD-1^+^ C-X-C chemokine receptor type 5 (CXCR5)^−^ CD4^+^ Th cells [60], and circulating T follicular helper (cTFH) cells [61], by recruiting B cells, play a humoral immune function to promote longer survival. Additionally, tumor-infiltrating follicular regulatory T (TFR) cells, which have been found to have high inhibitory capacity in multiple tumors, can reduce patient survival, possibly because of the high expression of immune checkpoint molecules, which weakens the immunotherapy response [62]. B cells participate in antigen presentation. Studies have found that there is a B cell subset defined as antigen-presenting B cells (BAPCs) with CD86^high^ and CD21^low^ that interact with T cells to fight tumors [27]. Researchers have also defined different wetting patterns of TILs in TME, except the previous introductions about components of TLSs and their functions and gene signatures. According to the presence and infiltration sites of TLSs, T and B lymphocytes in the TME, TILs are divided into immune structured, immune excluded, immune dispersed, and immune desertic [16]. Based on the location and number of infiltrating lymphocytes, they may be classified as type A, type B, or type C [51]. Moreover, some scholars have scored their immunoreactivity using a semi-quantitative method, which divides them into three groups (scoring 0–3) [52].

## 3. Microorganisms and Cancer

### 3.1. Carcinogenic Microorganisms

In recent years, cancer microbiology has attracted the attention of many researchers. At present, there are 12 known carcinogens, including bacteria, parasites, and viruses [63]. *Helicobacter pylori* (*H. pylori*) can generate stomach neoplasms or mucosa-associated lymphoid tissue (MALT) lymphoma through NF-kB/RASAL2/*β-catenin*, signaling axis, and the macrophage NLRC5 signaling pathway [64,65]. In addition to carcinogenicity, it also affects the results of anti-PD-1 and anti-cytotoxic T lymphocyte-associated antigen-4 (CTLA4), possibly by inhibiting antitumor CD8^+^ T cells due to abnormal DC activation [66]. Except for bacteria, schistosomiasis and clonorchis sinensis can induce bladder cancer and cholangiocarcinoma, respectively [11]. Of course, the most common carcinogenic microorganisms are still viruses. The expression of viral oncogenes and the host immune response, including inflammation and damage, can lead to cell mutations that induce cancer. Human papilloma virus (HPV) infection is a common pathogenic factor in genital tumors and HNSCC. The most common subtype is HPV16. Vaccines against HPV infection have been widely used in clinical practice. Recent studies have shown that E2 and E5 can be used as vaccine antigens in addition to the classic pathogenic genes E6 and E7 owing to their ability to evoke specific CD8^+^ T cell responses [26,67]. The combination of Merkel cell polyomavirus (MCPyV) DNA and the cancer genome may be the etiology for most MCCs [68]. Epstein-barr virus (EBV) infection is significantly associated with nasopharyngeal carcinoma (NPC), gastric cancer, and lymphoma. It can establish lifelong latency in the host to evade the host immune response [69]. Not all viruses rely on oncogenes. Chronic infection with the hepatitis virus gradually results in cirrhosis and liver cancer. Recurrent tissue damage due to viral replication, integration, and immune inflammatory responses, as well as liver regeneration and mutations, are important predisposing factors for liver cancer [70]. Human immunodeficiency virus (HIV) infection increases cancer risk and reduces survival, possibly because HIV destroys lymphocytes that can inhibit tumor replication [71]. In summary, the relationship between carcinogenic microorganisms and cancer has been a concern for decades, but there are still many questions to be addressed. For example, viruses with oncogenes, such as HPV, can lead to the occurrence of cervical cancer, but some patients can heal themselves after infection, whereas others develop cervical intraepithelial neoplasia (CIN) or cancer.

It is noteworthy that carcinogenic microorganisms have a wide range of carcinogenic effects and act as antigens to activate the immune system against tumors. Studies have found that HPV status in HNSCC is different, and there is a significant difference among the immune spectra, which may be caused by an increase in CD4^+^ follicular helper T (T_FH_) cells and B cells infected by the virus, for which antitumor immunity is enhanced [72]. Infected patients have better prognosis and immunotherapy response than non-infected patients [37,55]. The mechanism of this dual interaction between microorganisms and cancer requires further investigation.

### 3.2. Other Microorganisms

Several microorganisms, although not clearly defined carcinogens, can cause cancer through synergism [73]. *Escherichia coli* (*E. coli*) can promote tumor progression by producing the toxic metabolite colitoxin [74]. *Fusobacterium nucleatum* (*F. nucleatum*) can mediate the activation of glycolysis and cell proliferation by upregulating specificity protein 1 (SP1) expression and selectively targeting specific enolase1-intronic transcript 1 (ENO1-IT1), suggesting that gut microbiota and metabolism interact during tumorigenesis [75]. *F. nucleatum* can also induce alpha-kinase 1 (ALPK1) to stimulate the NF-κB pathway, leading to the upregulation of intercellular adhesion molecule 1 (ICAM1) and metastasis of colorectal cancer (CRC) [76]. Except for digestive system tumors, a fixed value for *F. nucleatum* present in breast cancer can induce lymphocyte apoptosis through lectin Fap2, resulting in tumor growth [77]. In addition to flora, carcinogenic fungi have recently received considerable attention. Researchers have found that the relative abundance of *Malassezia* increased significantly in pancreatic cancer patients and accelerated tumor progression through the activation of mannose-binding lectin (MBL) [78]. Another study on pancreatic cancer showed that fungal flora can drive the secretion of IL-33 to recruit and activate Th2 and innate lymphoid cells 2 (ILC2), stimulate the secretion of IL-13, and ultimately promote tumor growth [79]. A recent pan-cancer analysis also revealed a broad link between fungi and cancer, such as *Blastomyces* in lung cancer and *Candida* in gastrointestinal tumors, which are associated with inflammation and metastasis, and *Candida* also predicts poor prognosis [80]. Intratumoral Phaeosphaeriaceae or related *Phaeosphaeria* genera are related to shortened progression-free survival (PFS) in ovarian cancer [81]. Capnodiales and the genus *Cladosporium* show a significant increase in metastatic melanoma patients who have no response after immunotherapy [81]. Notably, the interactions between fungi and bacteria are closely correlated with tumors. They can stimulate the occurrence of CRC by upregulating d-arginine and d-ornithine or stimulating the butyrate metabolic pathway, indicating its potential as a new biomarker [82].

In contrast, some microorganisms can exert a protective effect and inhibit tumor progression. Reuterin inhibits CRC growth by altering redox balance and metabolite exchange [83]. *Streptococcus thermophilus* can secrete β-galactosidase to activate oxidative phosphorylation, downregulate Hippo pathway kinase, promote cell cycle retardation, and promote tumor cell apoptosis, thus playing a tumor-suppressor role [84]. Bacteroides and Parabacteroides in *Rnf5^−^/^−^* Mice inhibited melanoma growth by affecting DC activation [85].

Furthermore, intestinal microorganisms are involved in tumor immune regulation through their metabolites. Polyamines can inhibit anticancer immunity by inhibiting the proliferation of lymphocytes and inducing the production of tumor-derived proteases, thereby enhancing the invasiveness of tumor cells. Lipoteichoic acid (LTA) inhibits antitumor immunity by promoting prostaglandin E2 (PGE2) production via overexpression of cyclooxygenase-2 (COX2) [86]. In contrast, short-chain fatty acids (SCFAs) increase antitumor activation of cytotoxic T lymphocytes (CTLs) and CD4^+^ effector T cells [25,87]. Huang et al. found that ginseng polysaccharides (GPs) gain an antitumor reaction to αPD-1 monoclonal antibody (mAb) by increasing intestinal flora metabolites, such as valerate [88].

## 4. TLSs in Microorganism-Associated Cancer

### 4.1. Virus-Associated

A study on MCC found no correlation between the presence or number of TLSs and MCPyV infection. Both are independent prognostic factors [37]. In a study of EBV-associated gastric cancer, TILs and TLSs were found to be independent prognostic factors for EBV-negative gastric cancer rather than EBV-associated gastric cancer [89]. PD-1^+^ CXCR5^−^ cells can be used as components of TLSs to improve the prognosis of EBV-associated NPC. In addition, in EBV-positive gastroesophageal adenocarcinoma (GOA), CD8^+^ T cells are mainly located in the tumor center rather than the margin and express programmed death ligand 1 (PD-L1) as highly as tumor cells [90]. HPV infection is a common cause of cervical cancer and HNSCC. A study on cervical cancer found that the number of TLSs was significantly correlated with HPV infection, whereas the formation was correlated with preferable prognosis [91]. In the study of HNSCC, HPV-specific immunocytes appeared in HPV^+^ patients, and they may both participate in TLS formation and increase survival through GC response and GC B cell—T_FH_ cell interaction [12,26,67,72]. A more pronounced soakage of TLSs, particularly CD8^+^ PD1^+^ T cells, was found in chronic hepatitis virus-infected hepatocellular carcinoma (HCC) tumors than in non-infected ones and was associated with increased CTL failure [92]. Hepatitis B virus (HBV) infection and intratumoral TLSs in intrahepatic cholangiocarcinoma are associated with good prognosis. HBV infection may counteract excessive myeloid inflammation and re-activate antitumor immunity. TLS-positive tumors enriched with T and B cells specifically downregulate G2M checkpoints, inflammatory responses, and epithelial–mesenchymal transition (EMT) pathways, while upregulating metabolic and peroxisome pathways, suggesting that immune activation is beneficial to the efficacy of ICIs [93].

### 4.2. Bacteria-Associated

The implantation of *Helicobacter hepaticus* (*H. hep*) can induce *H. hep*-specific T_FH_ cells and promote the maturation of TLSs adjacent to the tumor [94]. A recent study on muscle-invasive bladder cancer (MIBC) found that urinary pathogenic symbiotic bacteria, especially *E. coli* with inherent immunogenicity and tumor invasion ability, can stimulate specific T_FH_ and B-cell reactions. The density of T_FH_-like cells correlated with TLS formation and PFS. *E. coli*-specific T_FH_ and IgG can predict clinical outcomes with neoadjuvant pembrolizumab treatment [95]. Wong-Rolle et al. analyzed the bacterial burden of TME cells in lung cancer patients and found that tumor cells had the highest bacterial burden compared to TLSs and adjacent normal tissues, which was positively correlated with gene enrichment of multiple carcinogenic pathways such as Wnt/β-catenin, hypoxia, and angiogenesis [96].

**Figure 3:**
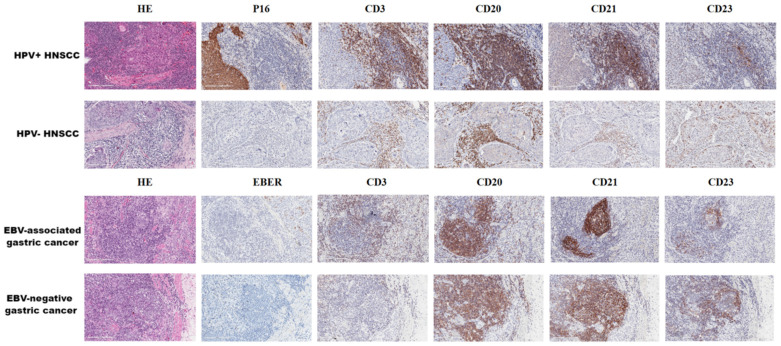
TLSs in microorganism-related cancer (×400). Representative images of tertiary lymphoid structures (TLSs) detected in formalin fixation with paraffin-embedding (FFPE) tumor sections via hematoxylin and eosin (HE) staining or immunohistochemistry (IHC) staining showing CD3^+^ T cells, CD20^+^ B cells, CD21^+^ follicular dendritic cells (FDCs), and CD23^+^ germinal centers (GCs). These tumor tissues contained human papilloma virus (HPV)-associated head and neck squamous-cell carcinoma (HNSCC) and Epstein-barr virus (EBV)-associated gastric cancer, and there was no significant difference in the morphological structure of TLSs. These histological images are original, unpublished images from the authors’ examination of tumors.

In summary, there are significant differences in the results of TLS studies on microorganism-associated cancers (Table 4). Most of the results support that microorganisms induce the formation and maturation of TLSs and correlate with good prognosis and immunotherapy response, which is consistent with the study of TLSs in pan-cancer [37,72]. TILs and other components of TLSs are involved in the composition of TME. Although the various types of cells in TLSs, especially TILs, have their own functions, it is necessary to consider TLSs as a special structure as a whole while emphasizing the spatial location and relationship between specific cells within TLSs [97,98] (Figure 3). For example, immature TLSs mainly consist of T cells, DCs, and follicles without GCs. In immature TLSs, high levels of PD-L1 on regulatory B (Breg) cells can cause T cell failure and inactivation, thereby inhibiting antitumor immunity [17]. In addition, Breg cells accelerate tumor growth by inhibiting interferon gamma (IFNγ) produced through CD8^+^ T cells [99] and also differentiate CD4^+^ T cells into the regulatory T (Treg) cell phenotype by producing transforming growth factor-beta (TGF-β) [100]. Microorganisms can promote the maturation of TLSs. After microbial infection, CXC-chemokine ligand 13 (CXCL13) produced by CD4^+^ T_FH_ cells can guide the migration of CXCR5^+^ B cells, promote the formation of GCs in TLSs, recognize antigens, and release antibodies to participate in humoral immunity. Simultaneously, B cells can communicate with CD8^+^ T cells to achieve their functions of antigen presentation and tumor killing [101]. IgD^−^ IgG^+^ CD27^−^ CD38^−^ CD20^+^ B cells in tumor-margin TLSs can also directly damage tumors by releasing granzyme B (GRzB) and TNF-related apoptosis-inducing ligand (TRAIL) [102]. Studies have shown that tumors with a mature TLS have a higher percentage of IgG-stained tumor cells, which are more likely to observe CD68^+^ macrophages and are significantly associated with a higher ICI response and longer PFS, indicating that macrophages may play an effector cell role through antibody-dependent cell-mediated cytotoxicity (ADCC) [34]. At the same time, tumor-associated macrophages in TME are also the main donors of PD-L1 [53]. Interestingly, more CD8^+^PD1^+^ T cells are present in microorganism-associated cancer [26]. In addition, activated antigen-presenting cells (APCs) can induce differentiation and expansion of PD-1^+^CXCR5^−^CD4^+^ Th-CXCL13 cell subsets through the TGF-β1 signaling pathway [60]. These results confirmed the correlation between microorganisms and the infiltration or exhaustion of CTLs and the existence of a cellular mechanism for blocking the response to PD-1, suggesting their potential as new biomarkers.

However, irrelevance between TLSs and microbes has also been reported. The reason for this difference may be related to the lack of literature, heterogeneity of the tumor, or mechanisms of action after different microbial infections. It is worth noting that microorganism-associated cancers usually have a better prognosis than uninfected cancers, while TLSs have a tumor-promoting effect in some tumors [103,104]. TLSs in clear-cell renal-cell carcinoma (ccRCC) are associated with poor prognosis and resistance to anti-angiogenic drugs after disease recurrence. This may be due to genetic changes in the PI3K-mTOR pathway components that produce many pro-inflammatory and pro-angiogenic cytokines. Although these abundant cytokines can recruit lymphocytes to form TLSs, excessive cytokines can also create an immunosuppressive environment [48]. Increased TLS abundance has been found in advanced gastric cancer [105], high-grade bladder cancer [106], and high-grade breast cancer [107], indicating that TLSs are associated with higher grade and stage. In addition, it was also detected that tumor cells infiltrated into TLSs and were associated with lymph node metastasis [106,107], indicating that TLSs may provide favorable conditions for lymph node metastasis of tumors.

## 5. Generation Mechanism and Induced Strategy of TLSs

### 5.1. Generation Mechanism of TLSs

TLSs are not ubiquitous in tumors, indicating that their occurrence requires specific conditions. Previous studies suggest that TLSs have the same occurrence process as SLOs because of their similar structures [15]. Inflammation-related molecules such as CXCL13 attract the aggregation of lymphoid tissue inducer (LTi) cells, which communicate with stromal cells through LTα1β2-LTβR, TNF-TNFR1, IL7-IL7R, and IL17-IL17R signaling pathways to promote the excretion of vascular endothelial growth factor C (VEGFC) to irritate HEV production. Then, the excretion of chemokines such as C-C motif chemokine ligand 19 (CCL19) and adhesion factors such as vascular cell adhesion molecule 1 (VCAM1) induce the homing of peripheral lymphocytes to the HEVs and control their entry into specific areas to form TLSs [5,108]. Th17 [109] and B cells [110] can replace LTi cells. Certain immune or stromal cells can secrete CXCL13 to act like lymphoid tissue organizer (LTo) cells, such as CD8^+^ T cells [58] in non-small-cell lung cancer (NSCLC), T_FH_ cells [111], and fibroblasts [112] in breast cancer. It is noteworthy that the secretion of chemokine CXCL13 can induce the expression of lymphotoxin-α1β2 (LTα1β2) on B cells or LTi cells through the CXCL13-CXCR5 axis and form a positive feedback loop, driving the expansion of stromal cells such as cancer-associated fibroblasts (CAFs) and TLSs [5].

**Figure 4:**
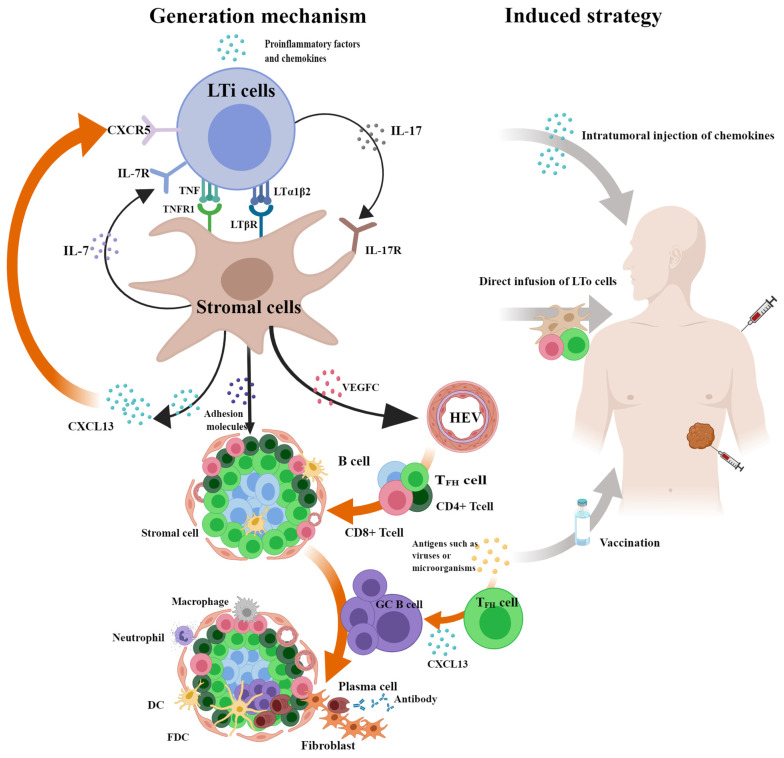
Generation mechanism and induced strategy of TLSs. Inflammation-related molecules such as CXC-chemokine ligand 13 (CXCL13) attract aggregation of lymphoid tissue inducer (LTi) cells which communicate with stromal cells through LTα1β2-LTβR, TNF-TNFR1, IL7-IL7R, and IL17-IL17R signaling pathways to promote the excretion of vascular endothelial growth factor C (VEGFC) to irritative high endothelial venules (HEV) production. Then, the excretion of chemokines and adhesion factors induce the homing of peripheral lymphocytes to the HEVs and control their entry into specific areas to form tertiary lymphoid structures (TLSs). It is noteworthy that the secretion of CXCL13 can induce the expression of lymphotoxin-α1β2 (LTα1β2) on B cells or LTi cells through the CXCL13-CXCR5 axis and form a positive feedback loop, driving expansions of stromal cells such as cancer-associated fibroblasts (CAFs) and TLSs. Except for the classical pathway, the mechanism of microbially induced TLS formation has been elucidated gradually. The formation and maturation of microbial-driven TLSs are closely related to the antigen recognition involved in follicular helper T (T_FH_) cells, which may be because T_FH_ cells can produce TLS-dependent CXCL13 to recruit B cells. Based on the mechanism of TLSs, it is feasible to induce TLSs through intratumoral injection or biomaterial implantation of cytokines and chemokines. Direct infusion of lymphoid tissue organizer (LTo) cells and intramuscular injection of vaccines may also promote TLS formation. The figure was drawn using the MedPeer program.

Except for the classical pathway, the mechanism of microbially induced TLS formation has been gradually understood. Studies have shown that oncolytic adenoviruses can promote vascular normalization and the formation of non-classical TLSs through stimulator of interferon genes (STING)-mediated DC activation [113]. The existence of GC in mature TLS suggests that antigen recognition may also be involved in driving TLSs. HPV and *H. hep* can induce significant increases in T_FH_ cells and the formation and maturation of TLSs in HNSCC and CRC, respectively, thereby increasing antitumor immunity [12,67,72,94]. In contrast, the number of TLSs decreased rapidly after the removal of pathogens in mice infected with influenza virus or in patients infected with *H. pylori* [13,114]. The above results indicate that the formation and maturation of microbial-driven TLSs are closely related to the antigen recognition involved in T_FH_ cells, which may be because T_FH_ cells can produce TLS-dependent CXCL13 to recruit B cells. A study on T_FH_ cells provides evidence that it can drive TLS formation, with researchers using a mouse model finding that TGF-β-mediated special AT-rich sequence binding protein 1 (SATB1) silencing leads to increased differentiation of T_FH_ cells, formation of intratumoral TLSs, and reduced tumor growth [115]. In conclusion, lymphotoxin signaling is not the only pathway that drives TLS formation (Figure 4). An in-depth understanding of the diverse origins of TLSs allows us to better understand the TLS heterogeneity detected in different tumors.

### 5.2. Induced Strategy of TLSs

Contraposing to the positive action of TLSs on protracting patient prognosis and strengthening immunotherapeutic effects, targeted induction of TLSs has received extensive attention and may become a new hope for future cancer treatment. A large number of animal experiments have proven that, according to the mechanism of TLSs, it is feasible to induce TLSs via intratumoral injection or biomaterial implantation of cytokines and chemokines such as lymphotoxin, CXCL13, and CCL21 [116,117]. Direct infusion of LTo cells into mice can also promote TLS formation [118]. Moreover, studies have indicated that PD-L1 blockade combined with anti-angiogenic therapy leads to HEV conversion and TLS formation [119]. After intramuscular injection of the HPV vaccine in patients with high-grade squamous intraepithelial lesions (HSILs) of the cervix, mature TLS can be formed in the vicinity of the original lesion, whereas no TLSs were observed in patients without the vaccine [120]. After intradermal injection of a tumor vaccine combined with cyclophosphamide in patients with pancreatic cancer, TLSs can be formed in tumors and prolong the survival time of patients. In patients without vaccine treatment, only a small amount of lymphocyte infiltration is observed [121]. These results indicate that treatment induction, vaccination, or a combination of both can drive the occurrence of TLSs (Figure 4).

Notably, decreased TLS density and GC deficiency were observed in patients with lung cancer treated with neoadjuvant chemotherapy (NC) and chemotherapy combined with steroids [47], indicating that TLS induction is not always positive or effective. Given the role of TLSs in autoimmune diseases, this negative outcome may be caused by the pro-inflammatory effects of mass-proliferating T and B cells in TLSs. Therefore, full account should be taken of the possible immunotoxicity during the therapeutic induction of TLSs, and reasonable assessments and controls should be made.

## 6. Conclusions

The role of TLSs in predicting prognosis and immunotherapy efficacy in cancer has been widely reported, but there is no standardized measurement guide. This study provides a reference for future research. Moreover, the induction strategy for the formation mechanism of TLSs is a new direction for future cancer treatment, such as cancer vaccines for microorganisms. The effect of microorganisms on cancer is dual, and the cancer-promoting functions of microorganisms have been widely demonstrated. However, the specific mechanism of its tumor suppressor effect, particularly the promotion of TLS formation, is unknown. In addition to the known communication between immune cells, whether they are also related to microbial metabolism has not been specifically reported. Future studies on TLSs in microorganism-related cancers should be supplemented by expanding the types and sample sizes of cancers and microorganisms, such as newly discovered fungi. Exploring the relationship between TLSs and microorganisms in cancer, especially the molecular network between them, will be helpful to fully understand the etiology and immune environment of cancer and empower the search for effective biomarkers and new treatments.

## Figures and Tables

**Table 1:** Common components of TLSs and corresponding indicators.

	Categories	Components	Immune Markers	Gene Signatures	Ref.
Immune cells (CD45^+^)	T cells (CD3^+^)	T_FH_ cells	CD4^+^, CXCR5^+^, CD40L^+^	CXCL13, CD200, FBLN7, ICOS, SGPP2, SH2D1A, TIGIT, PDCD1	[23]
		Th1 cells	CD45RO^+^	CD4, CCR5, CXCR3, CSF2, IGSF6, IL2RA, CD38, CD40, CD5, MS4A1, SDC1, GFI1, IL1R1, IL1R2, IL10, CCL20, IRF4, TRAF6, STAT5A	[24]
		Treg cells	CD4^+^ Foxp3^+^	NM	[15]
		CTLs	CD8^+^, CXCL-9,10,11,13	NM	[25]
		Dysfunctional/ Exhausted cells	CD8^+^PD1^+^	NM	[26]
	B cells (CD20^+^)	BAPCs	CD86^high^CD21^low^	NM	[27]
		GC B cells	CD38^+^BCL6^+^ AID^+^ Ki67^+^	NM	[16]
		Breg cells	CD19^+^	NM	[18]
		Plasma cells	CD38^+^/CD138^+^	TNFRSF17, IGJ	[28]
	Myeloid cells	DCs	CD83^+^DC-LAMP	NM	[16]
		pDCs	BDCA-2^+^ IRF7^+^	NM	[20]
		Macrophage	CD68^+^	NM	[22]
		Neutrophilic granulocyte	CD66b, Myeloperoxidase	NM	[21]
Non-immune cells (CD45^−^)	Stromal cells	FDCs	CD21^+^/CD23^+^	NM	[16]
		Fibroblast	PDPN^+^	NM	[18]
	Endothelial cells	HEVs	MECA79^+^ PNAd^+^	NM	[19]
Other molecular components	Chemokine and cytokine	CXCL13, CCL2, IL7, IL17	CXCL13, CCL21, IL7, IL17	CCL2, CCL3, CCL4, CCL5, CCL8, CCL18, CCL19, CCL21, CXCL9, CXCL10, CXCL11, CXCL13	[29]

NM, not mentioned; T_FH_ cells, T follicular helper cells; Th cells, T helper cells; Treg cells, regulatory T cells; CTLs, cytotoxic T lymphocytes; BAPCs, antigen-presenting B cells; GC, germinal center; Breg cells, regulatory B cells; DCs, dendritic cells; pDCs, plasmacytoid dendritic cells; FDCs, follicular dendritic cells; HEVs, high endothelial venules; CXCR, C-X-C chemokine receptor type; CXCL, CXC-chemokine ligand; IL, interleukin; CCL, C-C motif chemokine ligand; PD1, programmed cell death protein 1; TLR7, toll-like receptor 7; PDPN, Podoplanin; MECA79, mouse endothelial cell antigen-79; PNAd, peripheral node addressin; Ref., Reference.

**Table 2:** Comparison of TLS detection methods.

Method	Source	Target	Single Cell	Characterization	Quantification	Localization
HE	FFPE	Tissue	No	Yes	Yes	Yes
IHC/mIHC	FFPE	Protein	No	Yes	Yes	Yes
IF/mIF	Fresh tissues FFPE	Protein	No	Yes	Yes	Yes
FCM	Fresh tissues Peripheral blood	Protein	Yes	Yes	Yes	No
RNA-seq	Fresh tissues FFPE Peripheral blood	mRNA	No	Yes	Yes	No
scRNA-seq	Fresh tissues Peripheral blood	mRNA	Yes	Yes	Yes	No
ST	Frozen tissue FFPE	mRNA	No	Yes	No	Yes

HE, hematoxylin and eosin; FFPE, formalin-fixed and parrffin-embedded; IHC, immunohistochemistry; mIHC, multiplex immunohistochemistry; IF, immunofluorescence; mIF, multiplex immunofluorescence; FCM, flow cytometry; RNA-seq, RNA sequencing; scRNA-seq, single-cell RNA sequencing; ST, spatial transcriptomics.

**Table 3:** Detection, classification, and prognostic and predictive value of TLSs.

Tumor Type	Detection of TLSs	Presence and Prognosis	Quantity, Density, and Prognosis	Location and Prognosis	Differentiation and Prognosis	Composition and Prognosis	Predictive Value	Ref.
Melanoma	HE; IHC; mIF; scRNA-seq	Favorable	NM	Favorable (intra- tumoral)	No impact	NM	Gene signature associated with efficacy of ICB	[29]
Metastatic melanoma	HE; mIF	Favorable	NM	NM	No impact	Favorable (low fractions of CD21^+^ B cells)	NM	[46]
Lung squamous-cell carcinoma	HE; IHC; IF	NM	Favorable	Favorable	Favorable	NM	NM	[47]
HER2-positive breast cancer	HE; IHC	Favorable	NM	NM	NM	NM	NM	[39]
Endometrial cancer	HE; IHC	Favorable	No impact	No impact	Favorable	Favorable (number of CD20^+^ B cell)	NM	[41]
Clear-cell renal-cell carcinoma	HE; IHC	NM	NM	NM	NM	Poor (abundance of CXCL13^+^ CD8^+^ T cells)	NM	[36]
Kidney clear-cell carcinoma	HE; IHC	Poor	NM	NM	NM	NM	NM	[48]
Bladder cancer	HE; IHC	Favorable	NM	NM	NM	NM	NM	[48]
Stage II and III colorectal cancer	HE; mIF	NM	Favorable	NM	Favorable	NM	NM	[49]
Hepatocellular carcinoma	HE	NM	NM	Favorable (intra- tumoral)	Favorable	NM	NM	[50]
Pancreatic cancer	HE; IHC; IF	Favorable	NM	NM	Favorable	NM	NM	[40]
Gastrointestinal stromal tumors	HE; mIHC	Favorable	Favorable	No impact	NM	NM	NM	[43]
Glioblastoma	HE; mIF	NM	NM	NM	NM	Favorable (intratumoral densities of proliferating CD8^+^ T cells and higher CD8/CD4 ratios)	NM	[51]
Non-functional pancreatic neuroendocrine tumors	HE; IHC; mIF	Favorable	No impact	NM	NM	NM	NM	[44]
Epithelioid pleural mesothelioma	HE; IHC	Favorable	NM	NM	NM	Favorable (number of B cells)	NM	[52]
Epithelioid malignant peritoneal mesothelioma	HE	No impact	NM	NM	NM	NM	Associated with NC	[45]

HE, hematoxylin and eosin; IHC, immunohistochemistry; mIHC, multiplex immunohistochemistry; IF, immunofluorescence; mIF, multiplex immunofluorescence; scRNA-seq, single-cell RNA sequencing; CXC-chemokine ligand 13, CXCL13; ICB, immune checkpoint blockade; NC, neoadjuvant chemotherapy; NM, not mentioned; Ref., Reference.

**Table 4:** TLSs in microorganism-related cancer.

Microorganism	Tumor Type	TLSs and Prognosis	Classification of TLSs and Prognostic Value	Microorganisms and Prognosis	TLSs and Microorganisms	Ref.
MCPyV	MCC	Favorable	Presence	Favorable	NC	[37]
EBV	Gastric cancer	Favorable	Presence	NC	NM	[24]
EBV	NPC	Favorable	Composition (PD-1^+^ CXCR5^−^ CD4^+^Th cells)	NM	NM	[60]
EBV	GOA	NM	NM	NM	NM	[90]
HPV	CESC	Favorable	Presence	NM	PC	[91]
HPV	HNSCC	Favorable	Presence; Differentiation; Composition (B cell)	NM	PC	[12]
HPV	HNSCC	Favorable	Composition (CD4^+^ T_FH_)	Favorable	NM	[72]
HBV/HCV	HCC	Favorable	Composition (CD4^+^ T_CM_; CD20^+^ B cells)	NM	PC	[92]
HBV	ICC	Favorable	Presence; Location (intratumoral)	Favorable	NM	[93]
*H. hep*	CRC	NM	NM	NM	PC	[94]
*E. coli*	MIBC	Favorable	Composition (CD4^+^ T_FH_)	Favorable	NM	[95]

MCPyV, Merkel cell polyomavirus; EBV, Epstein-barr virus; HPV, human papilloma virus; HBV, hepatitis B virus; HCV, hepatitis C virus; *H. hep*, *Helicobacter hepaticus*; *E. coli*, *Escherichia coli*; MCC, Merkel cell carcinoma; NPC, nasopharyngeal carcinoma; GOA, gastroesophageal adenocarcinoma; CESC, cervical squamous-cell carcinoma; HNSCC, head and neck squamous-cell carcinoma; HCC, hepatocellular carcinoma; ICC, intrahepatic cholangiocarcinoma; CRC, colorectal cancer; MIBC, muscle-invasive bladder cancer; PD1, programmed cell death protein 1; CXCR5, C-X-C chemokine receptor type 5; Th, T follicular helper; T_FH_, follicular helper T; T_CM_, central memory T; NM, not mentioned; NC, no correlation; PC, positive correlation; Ref., Reference.
